# Supplementary material for: Identification of Genes Associated with Gut Microbiota Metabolites in Type 2 Diabetes Mellitus Complicated by Acute Cerebral Infarction Based on Bioinformatics and Mendelian Randomization
Source: Biomedicines. 2026 Oct 17;14(10):2371. doi: 10.3390/biomedicines14102371 (PMC13702449; doi:10.3390/biomedicines14102371)
Supplement: Supplementary file 1 [file biomedicines-14-02371-s001.zip › Figure S1/genus.RuminococcaceaeUCG004.id.11362 ACI.pdf]

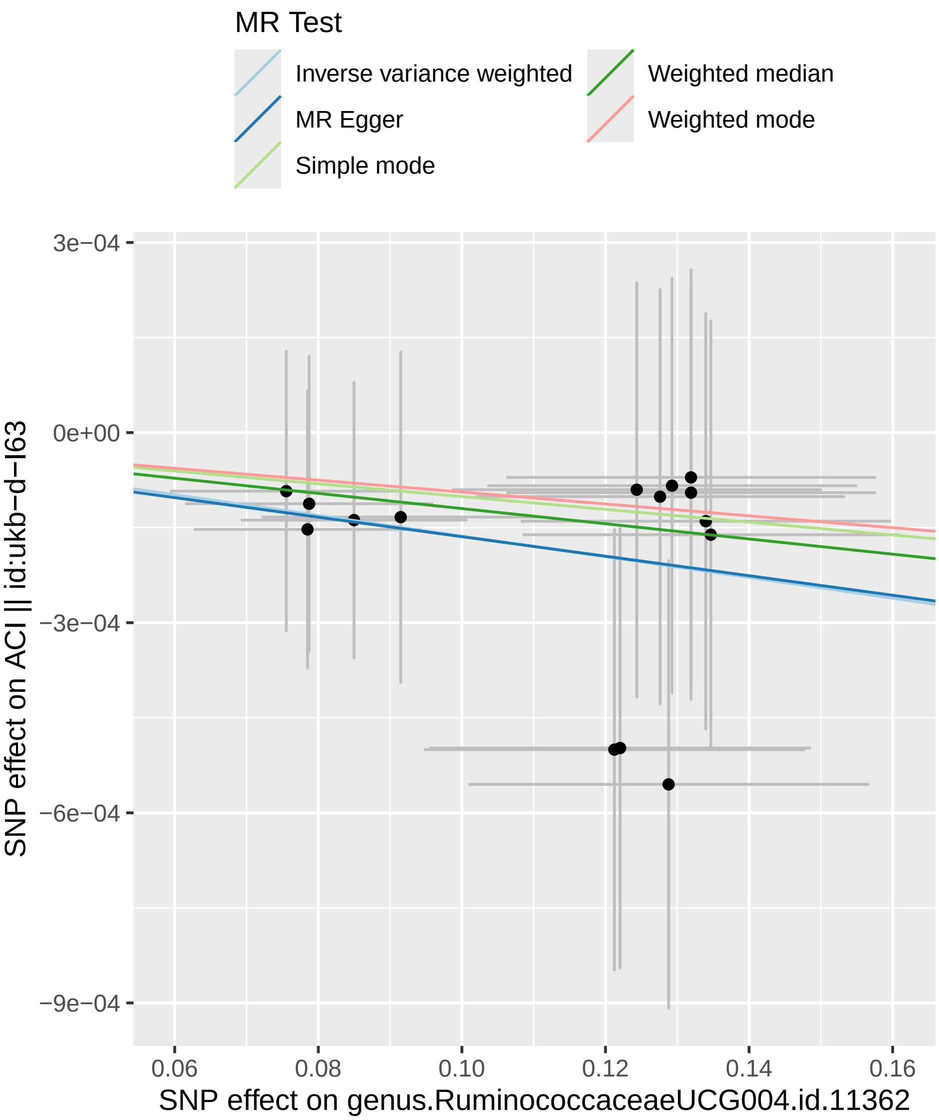
MR Test
Inverse variance weighted
MR Egger
Simple mode
Weighted median
Weighted mode
3e−04
0e+00
−3e−04
−6e−04
−9e−04
SNP effect on ACI || id:ukb−d−I63
0.06
0.08
0.10
0.12
0.14
0.16
SNP effect on genus.RuminococcaceaeUCG004.id.11362
